# The association between serum magnesium levels and hypertensive disorders of pregnancy

**DOI:** 10.4102/safp.v67i1.6140

**Published:** 2025-09-20

**Authors:** Naeera Abdul, Vinogrin Dorsamy, Chauntelle Bagwandeen

**Affiliations:** 1 Department of Laboratory Medicine & Medical Sciences, Faculty of Health Sciences, University of KwaZulu-Natal, Durban, South Africa; 2 School of Public Health and Nursing, Faculty of Health Sciences, University of KwaZulu-Natal, Durban, South Africa

**Keywords:** magnesium, pre-eclampsia, maternal health, HIV, obesity, South Africa

## Abstract

**Background:**

Evidence on circulating magnesium and hypertensive disorders of pregnancy in African populations is limited. We assessed between-group differences in serum magnesium and examined associations with early-onset pre-eclampsia and late-onset pre-eclampsia.

**Methods:**

We conducted an analytic cross-sectional study of 252 pregnant women sampled as four clinical groups: normotensive at a first antenatal visit, normotensive at term, early-onset pre-eclampsia, and late-onset pre-eclampsia. Serum magnesium was measured. Between-group differences were tested using one-way analysis of variance with contrasts. Associations with early-onset or late-onset pre-eclampsia versus normotensive groups were estimated using logistic regression adjusted for gestational age, body mass index, and human immunodeficiency virus status. We report effect sizes and 95 percent confidence intervals.

**Results:**

Serum magnesium was lower in normotensive women at term compared with those sampled at the first antenatal visit, consistent with physiological change across gestation. No comparable decrease was observed in early-onset or late-onset pre-eclampsia. The prevalence of hypomagnesaemia differed by group and was lowest at the first antenatal visit among normotensive women, while pre-eclampsia groups showed a divergent pattern. In adjusted models, obesity was positively associated with early-onset and late-onset pre-eclampsia, whereas human immunodeficiency virus status showed no significant association.

**Conclusions:**

In this South African cohort, cross-sectional comparisons demonstrate lower serum magnesium at term in normotensive pregnancies and a divergent profile in pre-eclampsia, supporting the hypothesis of altered magnesium regulation in hypertensive disorders of pregnancy. Results represent associations rather than causal effects and motivate future longitudinal research with repeated and intracellular magnesium measurements to clarify temporality and clinical relevance.

**Contribution:**

This article adds to the limited literature on Mg dynamics in pregnancy and highlights the need for population-specific strategies to reduce maternal mortality.

## Introduction

Magnesium sulphate is widely used in the management of pre-eclampsia (PE) to prevent seizures and progression to eclampsia, highlighting its established role in maternal care.^1^ However, the potential benefit of magnesium (Mg) supplementation earlier in pregnancy to prevent the development of PE remains underexplored. Understanding whether Mg insufficiency in early pregnancy contributes to the pathophysiology of PE is particularly relevant in high-risk populations, such as those in South Africa. As a country with persistently high maternal mortality rates, South Africa categorises maternal mortality as a distinct burden of disease, alongside infectious and non-communicable conditions.^2^ Therefore, investigating accessible and cost-effective interventions such as Mg supplementation could yield significant public health benefits. Magnesium is an essential micronutrient involved in vascular tone, endothelial function, blood pressure regulation and cellular function.^3,4^ During pregnancy, Mg supports foetal development and maternal well-being through its role in over 600 enzymatic reactions.^3,4^Physiological adaptations during gestation, such as haemodilution, increased renal excretion, and heightened foetal demands, contribute to a progressive decline in circulating Mg levels.^5^ These changes can predispose vulnerable individuals to hypomagnesaemia, particularly those with marginal nutritional intake, and may exacerbate the risk of adverse pregnancy outcomes, including hypertensive disorders of pregnancy (HDP).^6^

The South African context presents a unique setting to explore Mg’s role in pregnancy. The country paradoxically experiences a dual burden of malnutrition: undernutrition persists alongside a high prevalence of obesity, which affects nearly 46% of adult women.^7^ Obesity is a well-established risk factor for PE, and it is often associated with systemic inflammation and poor dietary diversity, including reduced intake of Mg-rich foods such as whole grains, nuts and leafy greens.^8,9,10^ In addition, South Africa has one of the world’s highest human immunodeficiency virus (HIV) prevalence rates, with approximately 4.8 million women affected.^11^ Both HIV infection and antiretroviral therapy (ART) have been linked to altered micronutrient metabolism, further complicating maternal nutritional status and pregnancy outcomes.^12^

Despite the established biological relevance of Mg in maternal physiology, there is a paucity of population-specific data on circulating Mg levels and their association with PE in South African women. This gap in the literature limits the ability to identify at-risk subgroups and develop targeted interventions.

This study aims to evaluate the relationship between serum Mg levels and HDP in a cohort of pregnant Black South African women. Specifically, we aim to: (1) compare Mg levels across different pregnancy groups, including normotensive and pre-eclamptic women; (2) assess the prevalence of hypomagnesaemia and its association with PE; and (3) identify potential predictors of PE, including obesity, HIV status and gestational age, in a population facing intersecting burdens of malnutrition, chronic disease and limited health care resources. By identifying modifiable nutritional risk factors such as Mg deficiency, this research contributes to the development of evidence-based public health strategies aimed at reducing maternal morbidity and mortality in South Africa and similar settings.

## Research design and methods

### Study design and setting

This was a hospital-based cross-sectional analytical study conducted at [*Prince Mshiyeni Memorial Hospital*], located on the east coast of South Africa. The study aimed to assess the association between circulating serum Mg levels and HDP including early-onset and late-onset pre-eclampsia (EOPE and LOPE), in a cohort of pregnant black South African women. Data collection occurred between June 2017 and January 2020.

### Study design justification

Although birth outcomes such as neonatal weight and gestational age at delivery were included in the analysis, the study maintained a cross-sectional design. Each participant was assessed once during pregnancy for clinical and biochemical data, and delivery outcomes were retrospectively extracted from medical records. No longitudinal follow-up or repeated measurements were performed. This approach aligns with cross-sectional designs commonly used in perinatal research that incorporate administrative or clinical outcome data.^13,14^

### Participants

A total of 260 pregnant women aged ≥ 18 years, attending routine antenatal care at the study hospital, were recruited using a convenience non-probability consecutive sampling. Only participants who provided informed written consent were included.

**FIGURE 1:**
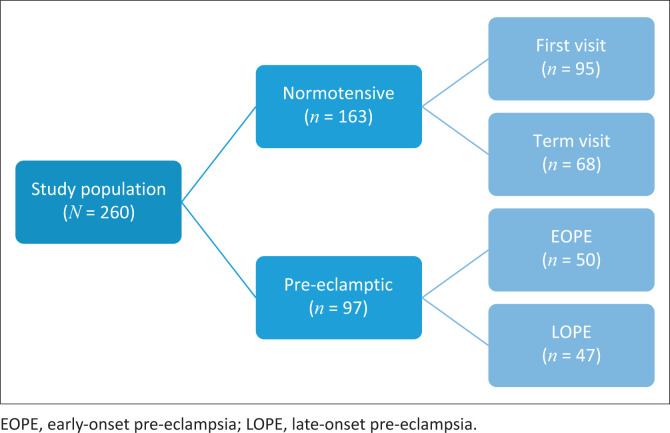
Flow chart illustrating the sample size per group.

Participants were categorised into four groups (Figure 1):

- First visit normotensive (First VisitNT): women attending their first antenatal visit between 12 and 28 weeks gestation, with normal blood pressure.
- Term normotensive (TermNT): women confirmed normotensive at term (≥ 37 weeks).
- EOPE: onset of pre-eclampsia before 34 weeks.
- LOPE: onset of pre-eclampsia at or after 34 weeks.

The diagnosis of pre-eclampsia was based on the International Society for the Study of Hypertension in Pregnancy (ISSHP) criteria: new-onset hypertension (≥ 140/90 mmHg on two occasions at least 4–6 h apart) after 20 weeks’ gestation, with one or more of the following: proteinuria, renal dysfunction, liver involvement, neurological features or haematological disturbances.^15^

### Inclusion and exclusion criteria

This study focused on pregnant black South African women who were 18-years-old and older. Participants who were willing to give informed consent who had a gestational age between 12 and 42 weeks were included. Participants with pre-existing chronic hypertension, diabetes mellitus or renal disease, who were diagnosed with gestational diabetes, had known multiple pregnancies or were using antihypertensives, Mg supplements, aspirin, warfarin or anti-inflammatory medications were excluded.

### Data collection and laboratory procedures

A structured data-collection form was used to obtain sociodemographic, clinical and obstetric history during antenatal visits. Trained nurses measured blood pressure, recorded gestational age and calculated body mass index (BMI) from measured weight and height. Gestational age was confirmed using a combination of last menstrual period (LMP) and first-trimester ultrasound when available. Baby weight was recorded at delivery using a standard neonatal scale.

### Sample collection

Venipuncture was performed to obtain blood samples, which were collected in BD Vacutainer® tubes. Serum was isolated via centrifugation (3000*g* for 12 min) and stored in Eppendorf tubes at −80 °C until analysis. Urine dipstick tests were conducted to confirm proteinuria.

### Magnesium sample collection and measurement

Venous blood was collected into BD Vacutainer® clot activator tubes and allowed to clot for 30 min before centrifugation at 3000*g* for 12 min. Serum was separated and stored at −80 °C in Eppendorf tubes until analysis. Serum Mg levels were quantified using atomic absorption spectrophotometry (Cobas Pro c 503, Roche Diagnostics, F. Hoffmann-La Roche Ltd). The process involved a 10-min colourimetric endpoint assay where Mg reacted with xylidyl blue in an alkaline buffer to form a purple complex. Concentrations were photometrically measured at 505/600 nm.^16^ Quality controls and calibrations were performed before analysis to ensure accuracy. Results were expressed in mmol/L. Hypomagnesaemia was defined as serum Mg < 0.66 mmol/L, based on National Health Laboratory Service (NHLS) reference values.

### Variables

- Primary exposure: Serum Mg concentration (continuous and categorical: normal vs. hypomagnesaemia).
- Primary outcome: Hypertensive status (normotensive, EOPE or LOPE).
- Covariates: Age, BMI (categorised as normal, overweight, obese per WHO), HIV status (positive or negative), gestational age at delivery.^7^

### Statistical analysis

Data were analysed using IBM® SPSS® Statistics Version 29.0 (IBM Corp., Armonk, NY). Continuous variables were tested for normality using the Shapiro–Wilk test. Descriptive statistics included means (±SD) for continuous variables and frequencies (%) for categorical variables. Baseline characteristics (e.g., age, BMI, gestational age) were summarised as means (±SD) for continuous variables and frequencies (%) for categorical variables. Following normality testing, an analysis of variance (ANOVA) was conducted to test the differences in Mg levels across groups and followed by post hoc Bonferroni comparisons. Chi-square tests were used to evaluate the association between Mg status (normal vs. hypomagnesaemia) and study groups. Multinomial logistic regression was conducted to identify predictors of PE (EOPE and LOPE) relative to normotensive groups, adjusting for covariates such as BMI, HIV status and gestational age.

Listwise deletion was used for cases with missing values on variables included in multivariable models. The extent of missing data for each variable was reported descriptively. No imputation techniques were used because of the relatively low proportion of missing data (< 10% for all key variables). A *p*-value < 0.05 was considered statistically significant.

### Ethical considerations

Ethical clearance was obtained from a sub-committee of the Biomedical Research Ethics Committee of a public university (BREC/00007005/2024). Written informed consent was obtained from all participants. Confidentiality was maintained by anonymising data and participation was voluntary. The study was conducted in accordance with the Declaration of Helsinki.^17^

## Results

### Study population and baseline characteristics

Out of 260 enrolled participants, 252 were included in the final analysis after excluding eight with incomplete records. Participants were categorised into four groups: First VisitNT (*n* = 94), TermNT (*n* = 67), EOPE (*n* = 49) and LOPE (*n* = 42). Table 1 summarises the baseline characteristics of each group.

**TABLE 1:** Baseline characteristics of study participants by group.

Characteristic	First VisitNT (*n* = 94)	TermNT (*n* = 67)	EOPE (*n* = 49)	LOPE (*n* = 42)
Age (years)	27.23 ± 5.56	28.46 ± 6.48	28.33 ± 6.14	28.88 ± 6.85
Gestational age at admission (weeks)	18.65 ± 5.52	37.94 ± 1.80	26.35 ± 5.40	37.05 ± 3.79
BMI (kg/m^2^)	29.05 ± 5.02	33.00 ± 6.17	33.60 ± 7.93	36.74 ± 8.56
Haemoglobin (g/dL)	11.31 ± 1.61	11.01 ± 1.57	11.04 ± 0.95	11.32 ± 1.26
Baby Weight (kg)	3.07 ± 0.58	3.17 ± 0.51	2.32 ± 0.73	2.81 ± 0.53
Magnesium Level (mmol/L)	0.78 ± 0.08	0.72 ± 0.10	0.78 ± 0.12	0.76 ± 0.09

Note: Values are presented as mean ± standard deviation.

BMI, body mass index; EOPE, early-onset pre-eclampsia; LOPE, late-onset pre-eclampsia; First VisitNT, first visit normotensive; TermNT, term normotensive.

The mean age of participants ranged from 27.2 ± 5.6 years (First VisitNT) to 28.9 ± 6.9 years (LOPE), with no significant age differences between groups. Body mass index was notably higher in the pre-eclamptic groups, with the highest mean BMI recorded in the LOPE group (36.7 ± 8.6 kg/m^2^), followed by EOPE (33.6 ± 7.9 kg/m^2^), TermNT (33.0 ± 6.2 kg/m^2^) and First VisitNT (29.1 ± 5.0 kg/m^2^). The TermNT group had the highest mean baby weight (3.17 ± 0.51 kg), whereas the EOPE group had the lowest (2.32 ± 0.73 kg).

### Serum magnesium levels across groups

Mean serum Mg levels differed significantly between groups (ANOVA, *F*(3,248) = 6.0, *p* < 0.001, η^2^ = 0.076). Among normotensive participants, a significant decline in Mg levels was observed between First VisitNT (0.78 ± 0.08 mmol/L) and TermNT (0.72 ± 0.10 mmol/L) groups (*p* < 0.001). This decline was not observed in the pre-eclampsia groups.

**FIGURE 2:**
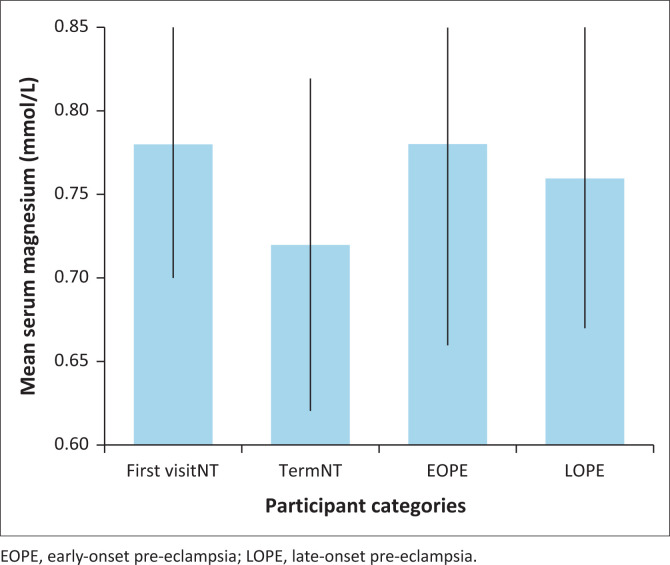
Mean serum magnesium levels (± standard deviation) by group.

Participants with EOPE and LOPE had mean Mg levels of 0.78 ± 0.12 mmol/L and 0.76 ± 0.09 mmol/L, respectively. No significant differences were found between either PE group and the First VisitNT group. However, EOPE participants had significantly higher Mg levels than those in the TermNT group (*p* = 0.003). The decline in serum Mg levels observed between the First VisitNT and TermNT groups was not present in the EOPE and LOPE groups, where Mg levels remained stable (Figure 2).

Magnesium levels declined significantly from First VisitNT to TermNT pregnancies. This decline was not observed in the EOPE and LOPE groups, where levels remained stable.

### Prevalence of hypomagnesaemia

Using the NHLS reference range (hypomagnesaemia defined as < 0.66 mmol/L), the overall prevalence of hypomagnesaemia was highest in the TermNT group (23.9%), followed by LOPE (9.5%), EOPE (12.2%) and First VisitNT (4.3%).

A chi-square test revealed a statistically significant association between Mg status (normal vs. hypomagnesaemia) and group classification (χ^2^(3) = 14.64, *p* = 0.002). Notably, hypomagnesaemia was associated with significantly reduced odds of pre-eclampsia (OR = 0.216, 95% CI: 0.065–0.721, *p* = 0.013), suggesting an inverse relationship between low Mg levels and PE in this sample.

### Human immunodeficiency virus across the groups

In the normotensive groups, HIV affected 51.1% and 40.3% of the FirstVisitNT and TermVisitNT participants, respectively, whereas 55.1% of the EOPE and 40.5% of the LOPE groups were affected by HIV. The HIV was not shown to be significantly associated with either hypertensive group.

### Predictors of pre-eclampsia

Multinomial logistic regression was used to identify independent predictors of EOPE and LOPE compared to normotensive participants. The model included BMI, gestational age at birth, serum Mg status and HIV status:

- Obesity was significantly associated with both EOPE (OR = 3.77, 95% CI: 1.35–10.53, *p* = 0.011) and LOPE (OR = 3.39, 95% CI: 1.36–8.47, *p* = 0.009).
- Gestational age at birth was strongly protective for EOPE (OR = 0.46, 95% CI: 0.35–0.61, *p* < 0.001).
- Magnesium status showed an inverse association with PE risk as observed earlier in the text.
- HIV status was not significantly associated with any hypertensive group.

## Discussion

This study examined the relationship between circulating serum Mg levels and HDP in a cohort of black South African women. Specifically, we sought to evaluate differences in serum Mg across normotensive and pre-eclamptic pregnancies, determine the prevalence of hypomagnesaemia and identify predictors of EOPE and LOPE. These objectives were addressed in the context of overlapping maternal health challenges in South Africa, including high rates of obesity, HIV infection and nutritional deficiencies.

The findings confirm that serum Mg levels decline significantly during the course of normotensive pregnancies, which is consistent with the expected physiological adaptations of gestation. In contrast, Mg levels in women with pre-eclampsia remained stable and did not exhibit the same decline observed in normotensive groups. This pattern was particularly notable in the comparison between TermNT and pre-eclamptic groups, suggesting that the regulatory mechanisms influencing Mg homeostasis may be disrupted in PE. This aligns with previous research proposing impaired renal excretion or endothelial dysfunction as mechanisms for altered Mg metabolism in pre-eclampsia.^18,19^

Hypomagnesaemia was most prevalent in the TermNT group and significantly less common among women with PE. Multivariate analysis showed that hypomagnesaemia was associated with a lower likelihood of both EOPE and LOPE. While this inverse association may appear counterintuitive, it is consistent with findings from studies in Ghana and South Africa, which have reported either no association or elevated Mg levels in women with HDPs.^13,14^ One possible explanation is that the stable or elevated Mg levels observed in pre-eclamptic women reflect underlying pathophysiological changes – such as endothelial damage or reduced renal clearance – rather than a protective effect of Mg itself. It is also plausible that Mg deficiency contributes to vascular risk only up to a threshold, beyond which systemic compensatory responses in pre-eclampsia alter circulating levels.

Obesity emerged as a strong independent predictor of both EOPE and LOPE in this study, consistent with literature highlighting the role of obesity-related inflammation and metabolic dysregulation in the pathogenesis of HDPs.^20,21^ Given the high prevalence of obesity among South African women (estimated at 46%), this finding underscores the urgent need for preconception and antenatal interventions that address nutritional status and weight management.^7^ Obesity may also mediate Mg imbalance through increased renal Mg loss, insulin resistance or altered dietary intake, although further studies are needed to clarify these pathways.

Human immunodeficiency virus status was not significantly associated with serum Mg levels or the risk of PE in our analysis. This finding contrasts with some prior studies suggesting a link between HIV infection, ART and micronutrient depletion.^22,23,24^ It is possible that the standardisation of ART regimens in South Africa and improved integration of antenatal HIV care have attenuated such associations. Alternatively, the sample size or limited granularity of ART-related data in this study may have obscured true effects.

Together, these findings support the hypothesis that Mg plays a complex role in pregnancy and HDP pathophysiology and also illustrate the fact that circulating Mg levels may be influenced by a variety of systemic factors. As such, low serum Mg alone may not be a reliable biomarker for predicting PE without considering co-factors such as gestational age, renal function, dietary intake and obesity status.

## Strengths and limitations

This study adds to the limited body of research on Mg dynamics in African populations, with particular relevance to public health settings burdened by nutritional and metabolic disorders. The use of a clearly defined cohort, standardised laboratory methods and multivariable adjustment are key strengths.

However, the findings must be interpreted in light of several limitations. Firstly, the cross-sectional design precludes causal inference, and the temporal relationship between Mg levels and HDP onset cannot be definitively established. Secondly, serum Mg does not reflect intracellular Mg, which may be more relevant to vascular function. Thirdly, dietary Mg intake, renal function and ART regimen details were not assessed, limiting insight into potential confounders or mediators. Lastly, restricting the study population to Black South African women, though intentional for population-specific analysis, limits generalisability to other groups.

## Conclusion

This study adds novel data on the profile of serum Mg levels across gestation in a South African population, revealing a divergence in trends between normotensive and pre-eclamptic pregnancies. The inverse association between hypomagnesaemia and PE challenges prevailing assumptions and invites further scrutiny into the regulatory mechanisms of Mg in pregnancy. As one of the few studies to report on this relationship in a resource-constrained African setting, the findings hold relevance for local antenatal screening strategies and the study also emphasises the importance of context-specific research in maternal health.
